# Monolayer Semiconductor Superlattices with High Optical Absorption

**DOI:** 10.1021/acsphotonics.4c00277

**Published:** 2024-06-17

**Authors:** Sara A. Elrafei, Lennart M. Heijnen, Rasmus H. Godiksen, Alberto G. Curto

**Affiliations:** † Department of Applied Physics and Eindhoven Hendrik Casimir Institute, Eindhoven University of Technology, 5600 MBEindhoven, The Netherlands; ‡ Photonics Research Group, Ghent University-imec, 9000Ghent, Belgium; § Center for Nano- and Biophotonics, Ghent University, 9000Ghent, Belgium

**Keywords:** monolayer semiconductors, heterostructures, superlattices, ultrathin-film absorption, molecular doping

## Abstract

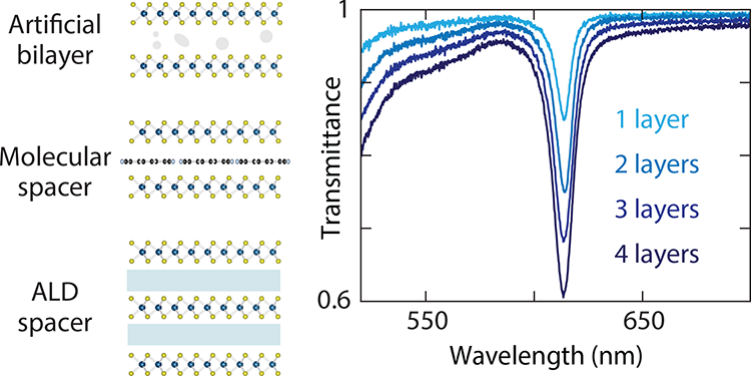

Optical absorption plays a central role in optoelectronic and photonic technologies. Strongly absorbing materials are thus needed for efficient and miniaturized devices. A uniform film much thinner than the wavelength can only absorb up to 50% of the incident light when embedded in a symmetric and homogeneous environment. Although deviating from these conditions allows higher absorption, finding the thinnest possible material with the highest intrinsic absorption is still desirable. Here, we demonstrate strong absorption by artificially stacking WS_2_ monolayers into superlattices. We compare three simple approaches based on different spacer materials to surpass the peak absorptance of a single WS_2_ monolayer, which stands at 16% on ideal substrates. Through direct monolayer stacking without an intentional spacer, we reach an absorptance of 27% for an artificial bilayer, although with limited control over interlayer distance. Using a molecular spacer via spin coating, we demonstrate controllable spacer thickness in a bilayer with 25% absorptance while increasing photoluminescence thanks to doping. Finally, we exploit the atomic layer deposition of alumina spacers to boost the absorptance to 31% for a 4-monolayer superlattice. Our results demonstrate that monolayer superlattices are a powerful platform directly applicable to improve strong light-matter coupling and enhance the performance of nanophotonic devices such as modulators and photodetectors.

## Introduction

Strong light absorption in ultrathin materials is sought after for light harvesting, photodetection, modulation, or sensing, both for free-space and guided-wave photonic devices. An ultrathin film is defined as a layer that is much thinner than the wavelength of light, thus resulting in a negligible change in the phase of light within the film.^1^ There exists, however, a theoretical fundamental limit to the absorption of light by such an ultrathin film when embedded in a homogeneous and symmetric refractive-index environment: the ultimate achievable absorption is 50% for a film by itself without additional help, a value that can be only met for a specific refractive index dependent on the film thickness.^2−4^ Such a limit is a manifestation of the underlying distribution of electric dipole transitions: when a dipolar sheet is surrounded by a homogeneous medium, perfect absorption is not possible for incident light from a single direction due to symmetry. Nevertheless, it is possible to surpass this absorption limit for ultrathin films by breaking the symmetry of the problem. One approach involves introducing a mirror positioned a quarter of a wavelength away from the thin layer. Such a Salisbury screen arrangement can lead to near-unity absorption.^4−8^ Nanostructure arrays such as metasurfaces can also enhance local fields for increased interaction with a thin film.^9−12^ Under interferometric excitation from both sides of the film, coherent perfect absorption is also possible under restricted conditions.^13^ Despite the possibility of exceeding 50% absorption by deviating from the conditions that define the validity of this limit for ultrathin films, it remains highly desirable to identify single materials that intrinsically absorb more light by themselves. Such stand-alone materials with high absorption would be more universally applicable than the extrinsic solutions described earlier, which rely on introducing other reflective materials, nanophotonic structures, or tailored illumination.

Various thin-film materials can indeed provide strong and sharp excitonic absorption. Molecular J-aggregates of specific organic dye molecules can exhibit a narrow absorption peak in the visible or near infrared. Thanks to their strong oscillator strength, they have enabled reaching the strong-coupling regime of light-matter interaction.^14−18^ Despite their remarkable properties, J-aggregates still have limited absorption, not reaching the theoretically possible maximum for ultrathin films by themselves, and lack versatility and tunability. Another promising class of strongly absorbing materials are two-dimensional crystals like graphene and transition-metal dichalcogenides. These materials are easy to assemble into complex heterostructures.^19^ As the bonding between monolayers and substrates occurs through van der Waals forces, lattice mismatch is not a limiting factor for creating heterostructures, unlike the case of conventional semiconductors grown epitaxially, such as Si or III–V materials. They also possess remarkable optical properties, with graphene exhibiting strong absorption at mid-infrared to terahertz frequencies.^20,21^ In contrast, its absorption in the visible and near-infrared ranges is limited to 2.3% for free-standing monolayers,^22,23^ and a large number of graphene layers is required for substantial absorption.^24,25^ Alternatively, monolayer semiconductors based on transition-metal dichalcogenides, such as WS_2_, exhibit uniquely high absorption coefficients despite being less than one nanometer thick. They absorb 5–10% of broadband sunlight, an order of magnitude higher than GaAs or Si of comparable thickness.^26,27^ Thanks to their strong and tunable excitonic resonances, they can serve as the basis for nanoscale optical components like metalenses, mirrors, spatial light modulators, and photodetectors for integrated photonic circuits.^28−32^ Their high oscillator strength and sharp exciton resonances are also promising for reaching the strong light-matter coupling regime^9,33−35^ and waveguiding.^36−38^

For such applications, where a strong excitonic resonance in a small material volume is required, semiconductor monolayers are still suboptimal due to their limited absorption. As a solution, several monolayers can be stacked into superlattices with alternating monolayers and dielectric spacers. This promising route has been recently explored to enhance the absorption and photoluminescence (PL) of MOCVD-grown monolayers using a gold reflector.^36^ Another recent study showed that bulk MoS_2_ with monolayer properties can be obtained by molecular intercalation and dedoping.^39^ Importantly, monolayer semiconductor stacks have been shown to improve the channel characteristics in stacked nanosheet transistors and could become relevant for industrial nanoelectronics and optoelectronics.^40−42^

Here, we demonstrate three methods to create high-quality superlattices of exfoliated WS_2_ monolayers to maximize ultrathin-film absorption. We focus first on structures containing two monolayers separated by a nanometric spacer to retain the optical properties of the constituent monolayers while keeping a minimal total thickness. We show enhanced absorption in artificial bilayers prepared by direct stamping, producing stacks without an intentional spacer. However, such structures are unstable, and further processing reduces the interlayer spacing, leading to decreased absorption. For increased robustness, we use a molecular spacer that also serves as a dopant, which yields increased PL as an added advantage. Finally, we produce higher-order superlattices by depositing spacers using atomic layer deposition (ALD). Although we demonstrate higher absorption in a 4-monolayer superlattice, the properties of the individual monolayers suffer degradation compared to the other fabrication methods, partially offsetting the scalability benefits. Our work demonstrates the potential of different monolayer superlattice configurations as a solid pathway to increase absorption using monolayer semiconductors for nanophotonic devices. As we use readily available fabrication methods, our results can be easily exploited in diverse applications requiring strongly absorbing ultrathin films, ranging from strong-coupling physics to integrated photonics.

## Results

### Maximizing Absorption with Monolayer Semiconductors

**Figure 1:**
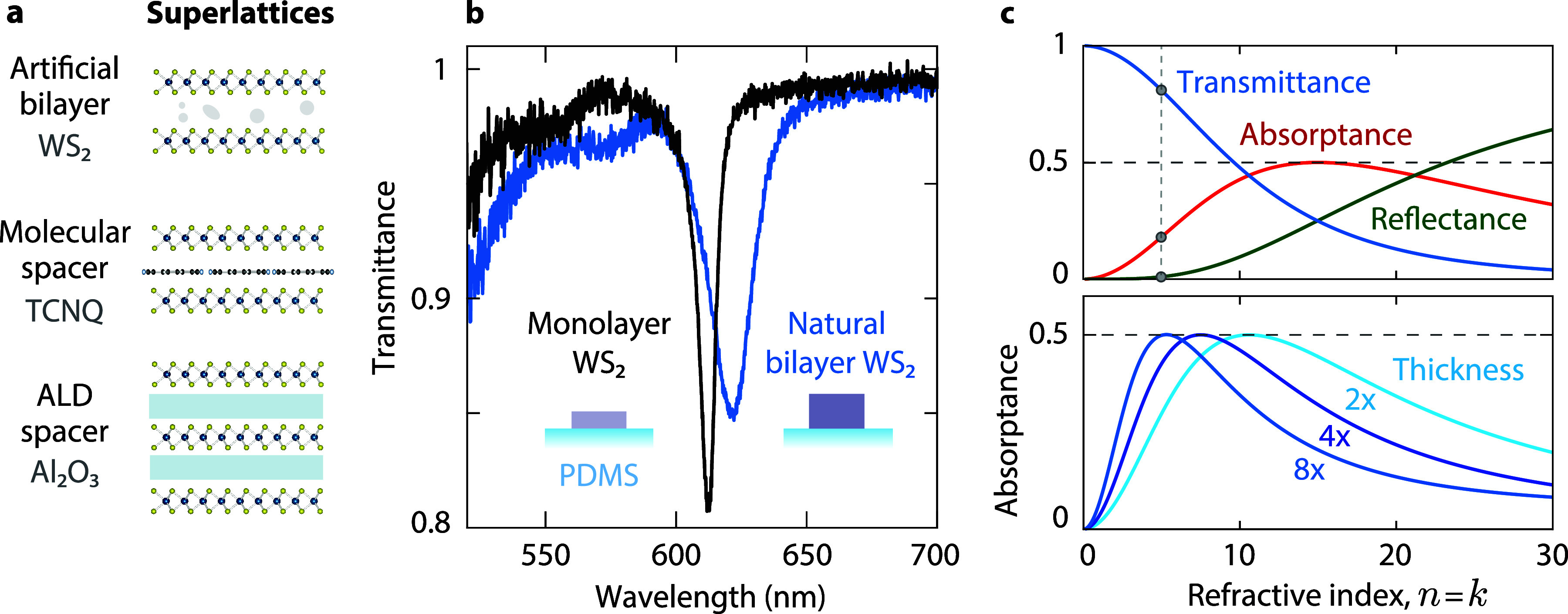
Stacking WS_2_ monolayers in superlattices for higher absorption. (a) Approaches for stacking monolayers with different spacers: no intentional spacer, molecular spacer, and atomic layer deposition spacer. (b) Transmittance of monolayer WS_2_ on PDMS (black), showing a stronger and narrower exciton peak than an exfoliated bilayer (blue). (c) Transfer-matrix method calculations illustrating the ultrathin-film absorption limit. Top: calculated transmittance, reflectance, and absorptance versus refractive index satisfying *n* = *k* for a 0.62-nm-thick layer in a symmetric PDMS environment (*n*_PDMS_ = 1.42) at λ = 613 nm. The refractive index of the WS_2_ monolayer at the A-exciton peak is close to 5 + 5*i* indicated by the vertical dashed line. Bottom: absorptance for increasingly thicker films in multiples of the monolayer thickness, showing maximum absorption for lower refractive indices along the *n* = *k* condition as the number of layers increases.

We chose monolayer WS_2_ as the absorbing material due to its record-high absorption coefficient, achieving peak absorptance surpassing J-aggregates and other transition-metal dichalcogenides (TMDCs) at room temperature.^15^ We start by mechanically exfoliating WS_2_ monolayers on a polydimethylsiloxane (PDMS) film attached to a glass slide. The PDMS substrate provides the best starting point known for WS_2_ at room temperature: a high peak absorption, narrow exciton line width, and high quantum efficiency.^43^ Compared to alternative substrates such as SiO_2_ on Si or glass, PDMS results in higher absorption and more uniform properties over the monolayer (Supporting Section S1). The transmittance spectrum of monolayer WS_2_ on PDMS exhibits an exciton resonance with a transmittance contrast of 16.9% and a transmittance minimum of 81.3% (Figure 1b and Supporting Section S2 for reflectance and absorptance). To determine the transmittance contrast, we first identify the baseline transmittance at wavelengths to the red of the excitons and compare it to the transmittance minimum at the A-exciton dip. The transmittance contrast is calculated as the difference between both values divided by the baseline. The main excitons in the transmission spectrum at wavelengths of 612.4 and 515.8 nm are referred to as the A and B excitons, respectively, along with a smaller dip due to the first excited state of the A exciton (*n* = 2).^44^ Although monolayer WS_2_ already offers remarkable absorption, an ideal ultrathin material with an optimal refractive index could still theoretically reach 50% absorptance in a symmetrical and homogeneous environment.

As a first candidate for improved absorption over the monolayer case, one might consider using a bilayer crystal. We compare a monolayer and a natural bilayer in Figure 1b, where the natural bilayer exhibits a lower transmittance contrast of around 14.1% with a transmittance minimum of 84.8% and a significantly broader line width than the monolayer (54 and 38 meV, respectively). The line width, γ, is defined as the full width at half-maximum of an excitonic absorption band (Supporting Section S3), and it is affected by interaction and decoherence mechanisms within the material. The higher absorption and narrower line width in a monolayer than a bilayer can be attributed to the absence of interlayer coupling, the reduced dielectric screening, and the enhanced radiative rate of excitons in the monolayer.^6^ Compared to the monolayer, these results indicate that natural bilayers or multilayers are not viable for maximizing absorption with the narrowest possible line width. Instead, we turn to artificial structures by purposefully stacking multiple monolayers into superlattices. We can maximize absorption in a superlattice with minimal thickness by maintaining a controllably low interlayer coupling. Using a short but sufficient spacer guarantees that desirable monolayer properties like a strong oscillator strength are not substantially modified in a superlattice, while still resulting in a very thin film useful for nanophotonic applications. We will demonstrate that this approach effectively enhances absorption by preserving the direct bandgap character of the monolayers, thus facilitating the accumulation of a strong excitonic response.

We adopt the transfer-matrix method to understand how light interacts with atomically thin semiconductors and their superlattices (Supporting Section S3). Given the thickness and complex refractive index of each layer, *ñ* = *n* + *ik*, we calculate the transmittance (*T*), absorptance (*A*), and reflectance (*R*) for thin-film stacks surrounded by two semi-infinite media under plane-wave illumination at normal incidence.^4^ First, we investigate a 0.62-nm-thick film, corresponding to a single WS_2_ monolayer but with variable refractive index, surrounded by PDMS (Figure 1c, top). We sweep the refractive index along equal real and imaginary parts of the refractive index (*n* = *k*), which is a necessary condition to reach the absorption limit for ultrathin films.^3,6,45^ We find indeed an absorptance maximum reaching 50% with transmittance and reflectance equal to 25%. Nevertheless, maximum absorption occurs when *n* = *k* ≈ 15 for a thickness equivalent to a single monolayer, which is not feasible in realistic optical materials. Our WS_2_ monolayers exhibit refractive indices close to *n* = *k* ≈ 5 (Figure 1c, dashed vertical line). As the film thickness increases to multiples of a monolayer, the requirement for maximum absorption shifts to lower values of the refractive index (Figure 1c, bottom). We predict thus that it could be possible to reach the ultrathin-film absorption limit using a realistic refractive index corresponding to monolayer WS_2_ but with a hypothetical film thickness equivalent to 8 monolayers. This estimate serves as a guide to illustrate the required quantity of material with monolayer-like properties required to theoretically achieve 50% absorption in superlattices.

### Stacking Monolayers into Artificial Bilayers

**Figure 2:**
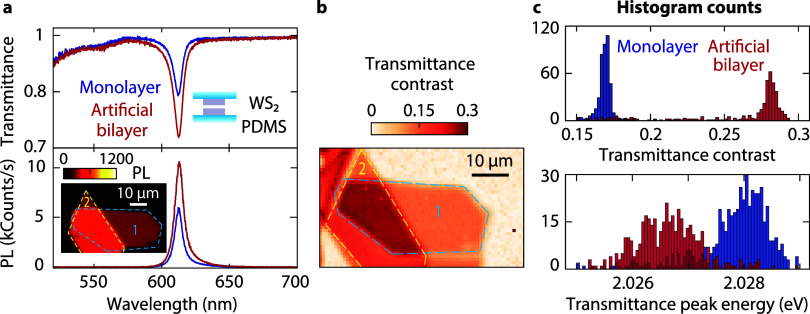
Increased absorption in an artificial WS_2_ bilayer consisting of two stacked monolayers. (a) Comparison of a WS_2_ monolayer (blue) and an assembled bilayer (red) in a symmetric polymer environment. Inset: PL map with higher luminescence on the overlap area between monolayers 1 and 2 (blue and yellow outlines). (b) Hyperspectral image of the same bilayer using transmittance contrast at the A-exciton peak energy. (c) Histograms obtained from hyperspectral transmittance imaging of monolayer and artificial bilayer areas. Top: transmittance contrast at the A-exciton peak. Bottom: A-exciton peak energy.

As the first step toward superlattices with multiple monolayers, we investigate artificial bilayers consisting of two stacked monolayers. To fabricate them, we deposit a WS_2_ monolayer directly on top of another monolayer based on a dry stamping method using PDMS (see Methods section). The resulting artificial bilayer is encapsulated in PDMS (Figure 2a, inset). Van der Waals forces bind the stack together, and the interlayer space contains air and impurities introduced during the fabrication process.^46−49^ Such an artificial bilayer shows a transmittance contrast of 28.7% with a transmittance minimum of 70.0% and a small increase in line width from 25.7 to 27.6 meV compared to the constituting monolayers (Figure 2a, top). When reflection is taken into account, we find an absorptance of 27% (Supporting Section S2). The PL intensity of the artificial bilayer roughly doubles the monolayer value (Figure 2a, bottom), which points to the possible absence of interlayer coupling as the origin of the preservation of the monolayer exciton quality in artificial bilayers. We observe an increase in PL line width from 20.7 to 23.2 meV and a small redshift, likely due to the higher permittivity of the surroundings of both monolayers due to the presence of the other monolayer, the additional PDMS superstrate, and impurities introduced in the stamping process.

We exploit hyperspectral imaging to quantify the spatial homogeneity of the exciton properties over an artificial bilayer. We record a spectrum at every point of a specified sample region to obtain statistics of several exciton properties in each area. Using hyperspectral transmission images, we retrieve the energy, line width, and transmittance contrast at the A-exciton peak (Supporting Section S4). There is a consistent rise in transmission contrast from 17% for a monolayer to 28% for an artificial bilayer. Furthermore, mapping shows the uniformity of the monolayer and artificial bilayer regions (Figure 2b), as confirmed by histograms with almost no overlap in transmittance contrast for the monolayer and artificial bilayer (Figure 2c). Additionally, we quantify a red shift in peak energy of approximately 1.5 meV.

**Figure 3:**
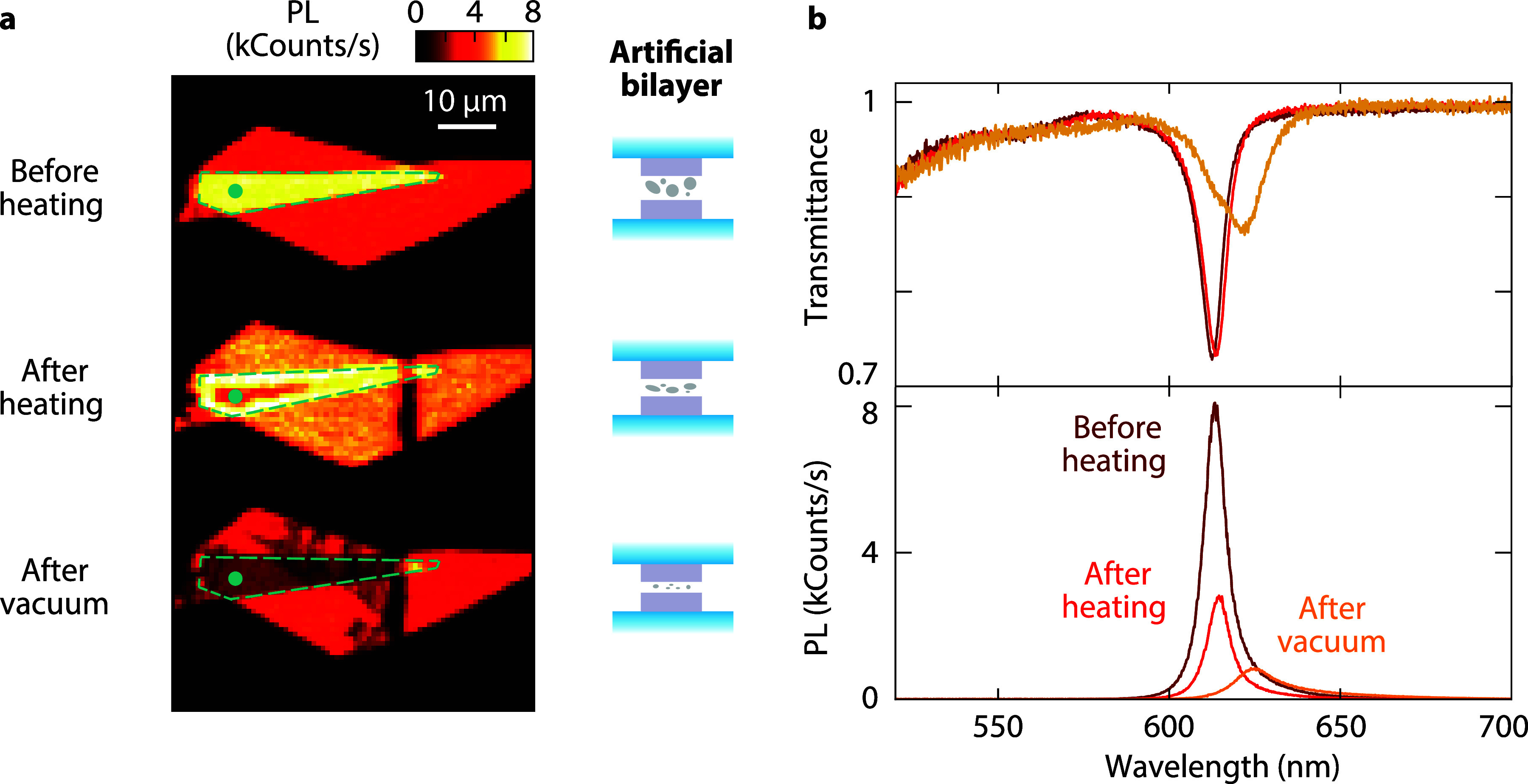
Control of spacer thickness in an artificial bilayer. (a) Confocal PL maps of stacked monolayers at different stages of closing the interlayer gap upon thermal annealing and vacuum storage. (b) Corresponding transmittance and PL spectra. After such treatments, the artificial bilayer area is quenched with spectra similar to those of a natural bilayer.

Although this method of producing bilayers is simple and already yields high absorption, we show next that the interlayer spacing is not fully reliable. We investigate the variability of the gap between the stacked monolayers. The stacks display consistent optical properties despite their random relative orientations, suggesting the presence of a small unintentional gap. This gap is likely filled with bubbles, exfoliation residue, and impurities from the transfer process. To reduce and control this spacer, we investigate the effect of applying heat and vacuum on a bilayer stack. Initially, the monolayers are nearly perfectly decoupled, doubling the PL intensity (Figure 3a, top). However, after heating the artificial bilayer at 80 °C for 20 min, interlayer interaction starts to appear, causing a PL gradient in the central area of the bilayer (Figure 3a, middle). As the temperature increases, the transmittance and PL spectra are slightly red-shifted, while contrast diminishes. As the gap closes further, the PL intensity decreases significantly, indicating a transition from weak coupling to strong interaction and leading to changes in the optical properties. After storage in vacuum, the spacer finally collapses (Figure 3a, bottom): the artificial bilayer starts resembling a natural bilayer with quenched PL and lower transmittance contrast, accompanied by a broader line width increasing from 23.0 to 78.2 meV (Figure 3b).

### Molecular Spacers between Two Monolayers

**Figure 4:**
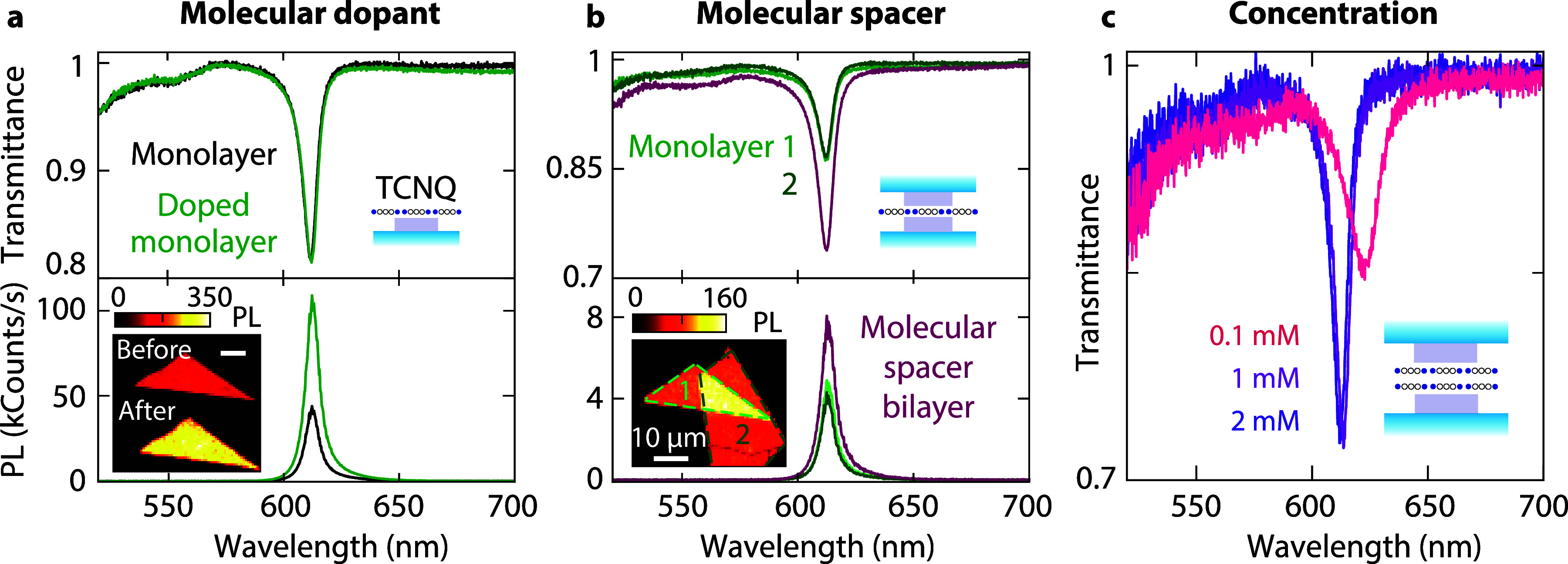
Molecular spacer bilayer with controlled interlayer distance. (a) WS_2_ monolayer with and without TCNQ doping: transmittance and PL spectra. Inset: PL maps illustrating the emission enhancement of a monolayer before and after doping with a TCNQ concentration of 1 mM. (b) Transmittance and PL spectra of an assembled WS_2_ bilayer with molecular spacer using a TCNQ concentration of 1 mM. Such a bilayer exhibits higher absorption and PL intensity than the monolayer. (c) Effect of molecular spacer concentration on the transmittance contrast.

To reliably maintain the desired monolayer properties by setting a minimum gap distance, we incorporate an intentional spacer layer. First, we employ a molecular spacer to control the interlayer coupling more precisely than in our previous artificial bilayers by introducing tetracyanoquinodimethane (TCNQ) molecules between the two monolayers. Interestingly, the adsorption of TCNQ molecules also induces charge transfer and provides *p*-type doping. It brings the *n*-type WS_2_ monolayer closer to an intrinsic semiconductor and increases its PL quantum efficiency without significantly altering the exciton energy and line width.^50−53^ We prepare the molecular spacer by spin coating a WS_2_ monolayer on PDMS with TCNQ in methanol (see Methods section). We focus first on a single monolayer and compare the transmittance and PL spectra of the monolayer with and without TCNQ doping. Using a TCNQ concentration of 1 mM, we observe an increase in the PL intensity of the monolayer, approximately three times higher than before doping (Figure 4a). As doping does not significantly change the transmission spectrum of a monolayer, these results verify the potential of TCNQ as an advantageous molecular spacer in superlattices.

We fabricate a bilayer with a molecular spacer by stamping an undoped monolayer on top of a doped monolayer. For a fair comparison with the previous artificial bilayers, we applied heating at 75 °C for 15 min and vacuum to ensure better contact between the layers. The molecular spacer yields a bilayer transmittance contrast of 28% with a transmittance minimum of 74% (Figure 4b), corresponding to approximately 25% absorptance (Supporting Section S2). In addition, it provides a 1.5-fold enhancement in PL intensity of the molecular spacer bilayer compared to a doped monolayer. We attribute the less than 2-fold increase to the sharing of the molecular dopant layer between both monolayers. The monolayer and bilayer areas are highly homogeneous without internal PL features (Figure 4b, inset).

We also show the dependence of the exciton properties in a molecular spacer bilayer on the TCNQ concentration. Spin coating with different concentrations corresponds to different molecular layer thicknesses: based on atomic force microscopy of a molecular film deposited on quartz, we estimate that TCNQ concentrations of 1 and 2 mM correspond to spacers of approximately 1 and 2 nm, respectively. Our results indicate that increasing the spacer thickness between two monolayers using a higher molecular concentration enhances the transmittance contrast and reduces the line width of the A exciton (Figure 4c). The line widths are 50.9, 26.3, and 25.8 meV for 0.1, 1, and 2 mM concentrations, respectively. The A-exciton peak also shifts to the blue. At a concentration of 0.1 mM, the monolayers interact strongly due to an insufficient spacer thickness, causing quenching and spectral broadening reminiscent of a natural bilayer. In summary, using a molecular spacer provides dual benefits for the optical properties of stacked monolayers: it enhances PL while facilitating improved light absorption. Despite the clear potential of molecular spacers in optimizing the optical properties of stacks of layered materials, our fabrication method cannot be easily scaled to thicker superlattices to further increase absorption.

### Scalable Superlattices through ALD of Spacer Layers

**Figure 5:**
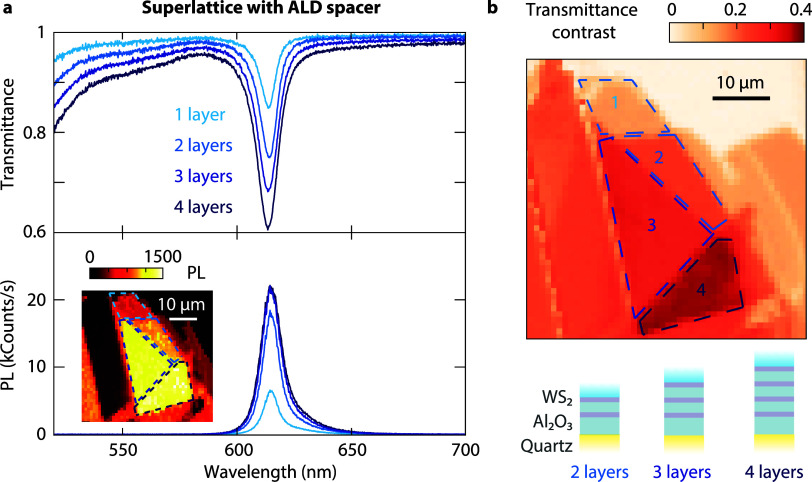
Superlattice with alternating WS_2_ monolayer and Al_2_O_3_ layers deposited through atomic layer deposition. (a) Transmittance and PL spectra of superlattices with an increasing number of WS_2_/Al_2_O_3_ unit cells. (b) Map of transmittance contrast variations for different superlattice thicknesses. Schematic illustration of the assembled layers for 2, 3, and 4 WS_2_ monolayers. The stacks are protected with PDMS capping.

The artificial and molecular spacer bilayers benefitted from a PDMS substrate to maximize exciton absorption. Using a rigid substrate instead of a flexible polymer film typically results in lower monolayer absorption, but it also enables compatibility with other spacer deposition methods that facilitate the scalability of monolayer superlattices. Next, we use ALD to grow alumina (Al_2_O_3_) spacer layers (see Methods section). We deposit 50 nm of Al_2_O_3_ on amorphous quartz as a substrate for the first WS_2_ monolayer. We prepare superlattices using the PDMS dry transfer method with subsequent spacer layers deposited using ALD. For a bilayer stack with a 2-nm-thick spacer, we observe a transmittance contrast of 23.9% (Figure 5a). We attribute the reduction compared to the previous fabrication methods to the presence of a different substrate, strain, and damage during the ALD process (Supporting Section S5).^43,54^

We fabricate thicker superlattices with 3 and 4 alternating WS_2_ monolayers and spacers. The transmittance contrast increases with the number of layers, as seen in a hyperspectral transmittance image at the A-exciton peak (Figure 5b). The transmittance contrasts for 1-, 2-, 3-, and 4-monolayer superlattices are 14.2, 23.9, 30.6, and 36.4%, respectively. The contrast for the B exciton to the blue of our spectral range also grows with the number of monolayers (Figure 5a). Relying solely on transmission as an indicator for absorption is insufficient because reflection cannot be neglected for structures with increased absorptance (Figure 1c and Supporting Section S2). Taking into account reflection, the absorptances of the 1-, 2-, 3-, and 4-monolayer superlattices are 13.8, 21.6, 26.6, and 30.8%, respectively.

The PL of a superlattice initially increases with the number of layers, but the peak intensity does not grow linearly for additional layers (Figure 5a, bottom). Although we kept a low deposition temperature of 100 °C to minimize damage, the additional defects due to the ALD process and trapped impurities introduced by stamping negatively affect the quantum efficiency of each monolayer cumulatively. Consequently, emission saturates for 3- and 4-monolayer superlattices (Figure 5a, inset). To conclude, building superlattices with ALD spacers provides a scalable route to increased absorption and brighter light emission. However, the spacer deposition process needs further optimization to fully take advantage of this approach to reach 50% absorptance with the thinnest possible superlattice.

## Conclusions

We demonstrated the efficacy of superlattices formed by stacking multiple WS_2_ monolayers to maximize light absorption in ultrathin films. Superlattices provide higher absorption than high-quality exfoliated monolayers and bilayers while preserving the narrow exciton line width and emission quantum efficiency of single monolayers. We compared three approaches based on direct stacking without an intentional spacer, with a molecular spacer, and with an alumina spacer. Creating an artificial bilayer through simple stacking led to a substantial increase in transmittance contrast to 30% from 17% for a monolayer, albeit with limited control over gap thickness. Introducing a molecular spacer using TCNQ proved highly effective, offering controllable thickness through varying molecular concentrations. A bilayer stack with a molecular spacer allowed us to reach 28% transmittance contrast. Additionally, this molecular spacer also increases PL by acting as a dopant. Finally, we used ALD of insulating spacers to increase the number of monolayers and boost the transmittance contrast to 36%. Further fabrication optimization and scaling of these monolayer superlattices are still possible.

The versatility of our superlattices and the simplicity of the fabrication methods present compelling advantages for their incorporation into devices that would benefit from improved absorption. Our demonstration opens avenues for nanophotonic devices with superior light harvesting, emission, or modulation capabilities. Moreover, monolayer superlattices can be leveraged to easily improve light-matter interaction in the strong-coupling regime and waveguiding using surface exciton-polaritons compared to single monolayers. Beyond increased absorption, engineering superlattices to fine-tune the optical properties of semiconductor monolayers can enable nanoscale sensors and subnanometric rulers based on changes in exciton properties. Monolayer semiconductor superlattices offer thus solutions for efficient and miniaturized nano-optoelectronics and integrated photonics converging with future atomically thin electronics.

## Methods

### Preparation of Monolayer Superlattices

We mechanically exfoliate a bulk WS_2_ crystal (*n*-type doping, HQ Graphene) down to a monolayer using tape (SPV 9205, Nitto Denko) onto an optically transparent PDMS film (Gel-Pak PF-80-X4). We identify monolayer areas using a fluorescence wide-field microscope and transfer them to target positions using two *XYZ* stages. The PDMS film acts as a stamp for all-dry viscoelastic stamping^55^ used in the following three approaches to construct monolayer stacks.

#### Artificial Bilayer

The PDMS film, with the first monolayer on top, lies on a glass slide. We transfer a second monolayer carried by another PDMS film onto a target monolayer on the PDMS/glass substrate. We leave the top PDMS film on the stack to prevent unnecessary damage to the monolayers. After initial optical characterization of the as-deposited bilayer, we treat some samples to test the robustness of the interlayer gap. Initially, we subjected those samples to thermal annealing at 80 °C for 20 min on a hot plate in air to remove bubbles and impurities. Subsequently, we stored the sample in a vacuum desiccator with a VWR VP 86 diaphragm pump to ensure uniform layer-to-layer contact.

#### Molecular Spacer

The molecular spacer approach starts with a monolayer on a PDMS film supported on a glass slide. To create the molecular spacer, we use 7,7,8,8-tetracyanoquinodimethane (TCNQ, Ossila Ltd.). We prepare solutions with different molecular concentrations in methanol: 0.1, 1, and 2 mM. For example, we dissolve 4.1 mg of TCNQ powder in 20 mL of methanol for a concentration of 1 mM. To spin-coat a WS_2_ monolayer on a PDMS/glass substrate, we pipet 20 μL of TCNQ solution, followed by spinning for 1 min at 500 rpm. At this speed, the deposition produces a thin, flat, and homogeneous film^56−58^ with minimal damage to the monolayer semiconductor. The TCNQ molecules are expected to lie flat on the monolayer, facilitating their use as a controlled spacer. Next, we stamp a second monolayer on top of the TCNQ-coated monolayer. As in the case of the artificial bilayer, we include thermal annealing and vacuum treatment. We leave the PDMS superstrate covering the resulting molecular spacer bilayer.

#### ALD Spacer

For the monolayer superlattices based on ALD spacers, we transfer the monolayers from PDMS onto ALD-coated, amorphous quartz wafer pieces (Planoptik). We deposit the Al_2_O_3_ layers using a high-vacuum ALD system (FlexAL2, Oxford Instruments). Trimethylaluminum (TMA) and water vapor serve as precursor gases, and the deposition occurs with the chamber and table at 100 °C. Although it could produce higher-quality oxide films, higher deposition temperatures negatively affect the monolayers, resulting in quenching and the appearance of dark spots on the monolayer superlattices. After every monolayer transfer step in the superlattice construction, we remove the PDMS film by storing the sample under vacuum for 1 day (pressure in the mbar range). Vacuum storage guarantees good and homogeneous contact between the layers, further improved by heating at 70 °C for 10 min on a hot plate in air. Next, the sample cools down for 5 min. Then, the PDMS is peeled off carefully using sharp tweezers, leaving a new WS_2_ monolayer on the superlattice. This method of PDMS removal is gentle on the TMD, as evidenced by the consistent fluorescence lifetime measurements taken before and after the process.^43^ Note that during the final assembly step of the superlattice, we leave the PDMS as a superstrate for the top monolayer, which did not undergo ALD processing. The bottom monolayer, on the other hand, went through ALD processing 1, 2, and 3 times for the superlattice areas in Figure 5 with 2, 3, and 4 monolayers, respectively.

### Optical Characterization

We use a sample-scanning confocal microscope for transmission, reflection, and PL imaging and spectroscopy. The setup can also perform hyperspectral imaging by recording a spectrum at every point. For reflection and transmission measurements, we illuminate the sample with an incandescent white light source using Köhler illumination modules from the top or the bottom of the sample. For the bottom path used in transmission configuration, we use a microscope objective with adjustable cover-glass correction (Nikon CFI Plan Fluor ELWD 20×, NA = 0.45) to illuminate the sample through the supporting glass slide. The transmitted or reflected light is collected through the top objective (Nikon CFI Plan Fluor ELWD 40×, NA = 0.6), allowing for optical thickness correction of a possible PDMS top layer. After attenuation by a neutral density filter, light is coupled into a fiber with a core size of 50 μm. The signal is then guided into a spectrometer (Andor Shamrock 303i spectrograph with an Andor Newton 970 EMCCD camera cooled to −70 °C) to carry out spectroscopy or hyperspectral imaging, or to an avalanche photodiode (Micro Photon Devices, PDM50) for imaging.

For PL measurements, we use a continuous-wave laser at 532 nm (Cobolt Samba) with neutral density filters to control the power reaching the sample in the 1–100 μW range, depending on PL efficiency changes due to doping and quenching. The excitation laser is cleaned using a band-pass filter (Thorlabs, FLH532–4) and reflected toward the sample by a nonpolarizing beam splitter (Chroma, 21014 Silver Non-Polarizing 50/50 bs). It is focused on the sample by the same top objective, resulting in PL emission filtered using a long-pass filter (Thorlabs, FELH0550) and collected in epifluorescence configuration through the same optical path as the reflection measurements.

## Supporting Information Available

Comparison of WS_2_ monolayer on PDMS and on glass; transmittance, reflectance, and absorptance spectra; transfer-matrix method and the maximum absorption limit; hyperspectral analysis of different spacers; and effect of atomic layer deposition on the WS_2_ monolayer (PDF)
