# Supplementary material for: Monolayer Semiconductor Superlattices with High Optical Absorption
Source: ACS Photonics. 2024 Jun 17;11(7):2587–94. doi: 10.1021/acsphotonics.4c00277 (PMC11258785; doi:10.1021/acsphotonics.4c00277)
Supplement: Supplementary file 1 — ph4c00277_si_001.pdf [file ph4c00277_si_001.pdf]

Supporting Information

Monolayer semiconductor superlattices with high optical absorption

Sara A. Elrafei[1], Lennart M. Heijnen[1], Rasmus H. Godiksen[1], Alberto G. Curto[1,2,3*]

[1] *Department of Applied Physics and Eindhoven Hendrik Casimir Institute, Eindhoven University of Technology, Eindhoven, The Netherlands*

[2] *Photonics Research Group, Ghent University-imec, Ghent, Belgium*

[3] *Center for Nano- and Biophotonics, Ghent University, Ghent, Belgium*

* Corresponding author: Alberto.Curto@UGent.be

Contents (8 pages, 5 figures)

Supporting Section S1. Comparison of WS_2 monolayer on PDMS and on glass.

Supporting Section S2. Transmittance, reflectance, and absorptance spectra.

Supporting Section S3. Transfer-matrix method and the maximum absorption limit.

Supporting Section S4. Hyperspectral analysis of different spacers.

Supporting Section S5. Effect of atomic layer deposition on WS_2 monolayer

Supporting Section S1. Comparison of WS_2 monolayer on PDMS and on glass

The choice of substrate influences the properties of WS_2 monolayer. The substrate can introduce strain, affect charge transfer, modify the dielectric environment, and introduce surface roughness, all of which collectively impact the electronic and optical characteristics of the monolayer. In this Section, we conduct a comparative analysis of the optical properties of monolayer WS_2 on various substrates such as PDMS, and glass. Supporting Figure S1a (top) shows the normalized PL of the two samples. Ideally, one would compare the same monolayer on both substrates, but transferring a monolayer between

substrates is challenging as it causes damage. Instead, we used separate monolayers while ensuring they had comparable quality by examining their photoluminescence lifetime, which serves as an indicator of the overall exciton quality. We see a significant increase in linewidth and a redshift of around 12 meV for the glass substrate. This shift can be attributed to the higher permittivity of glass compared to PDMS, resulting in a lower permittivity contrast with the monolayer; as a result, stronger dielectric screening for the monolayer on glass leads to a decreased binding energy and a shift of the band gap to lower energy, an effect discussed by Raja *et al.* (Nat. Nanotechnol. 14, 832–837 (2019)).

In Supporting Figure S1a (bottom), we present the transmittance of WS_2 monolayers deposited on PDMS and glass substrates. The A-exciton peaks are at 613 nm (2.023 eV) for PDMS and 616 nm (2.013 eV) for glass, indicating an influence of the substrate on the peak position. The monolayer on the PDMS substrate exhibited a more pronounced transmittance contrast (0.17 compared to 0.10 for glass) and a narrower linewidth (23 versus 39 meV), suggesting that PDMS is a more suitable substrate for monolayer WS_2. This is expected, as glass provides a less clean and less homogeneous dielectric environment for the monolayer. Finally, we also compare the quality of PDMS and glass as substrates using hyperspectral PL imaging, with part of the results summarized in the histograms in Supporting Figure S1b. The redshift in peak energy, around 9 meV on average, and the increased linewidth, by around 6 meV, of the sample on glass are both clearly visible.

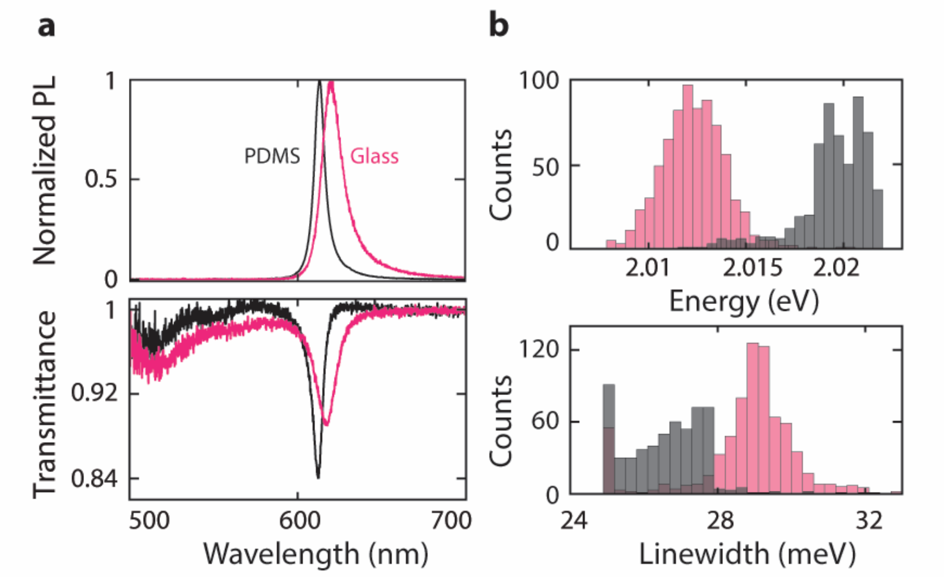

Supporting Figure S1 | Using PDMS as a substrate for WS_2 monolayer results in high absorption, narrow exciton linewidth, and ensures uniform properties compared to a glass substrate **a,** Comparison of the normalized photoluminescence and transmission spectra of a WS_2 monolayer on glass and PDMS substrates. **b,** Comparison of histograms obtained through hyperspectral imaging of the photoluminescence of monolayers on the same substrates.

Supporting Section S2. Transmittance, reflectance, and absorptance spectra

As part of our experiments, we measured transmittance and reflectance to determine the absorptance of three types of thin-film structures of monolayer WS_2, including air-spacer bilayers (or artificial bilayers), molecular-spacer bilayers, and Al_2O_3-spacer superlattices. We observed that reflectance played a small role in the optical response of monolayers and bilayers, which led us to refer primarily to transmission data for ease of comparison by other authors as it relies on a single measurement but serves as a good proxy for absorption. The low reflectance of monolayers and bilayers compared to peak transmittance contrast is indeed predicted by Figure 1c because the refractive index is too low to produce significant reflectance for such a small thickness. However, we show next that this simplified treatment is less applicable in the case of higher-order superlattices. We present the result of such optical measurements in Supporting Figure S2. The artificial bilayer exhibits a substantial improvement in transmission

contrast compared to a monolayer, accompanied by a slight increase in the reflectance spectrum from a reflectance peak of 1% to approximately 3.7%. Following the equation $A = 1 - R - T$, we computed the corresponding absorptance spectra, which results in an increase in the absorptance peak from 17% to 27%. For the molecular-spacer bilayer, where we employed TCNQ molecules as controlled spacers, we achieved a similar absorptance close to 27%. In the case of higher-order superlattices incorporating multiple Al_2O_3 layers using atomic layer deposition alternating with monolayer WS_2, the reflectance peak reaches approximately 9% for structures with four monolayers, which yields a maximum absorptance of 31%.

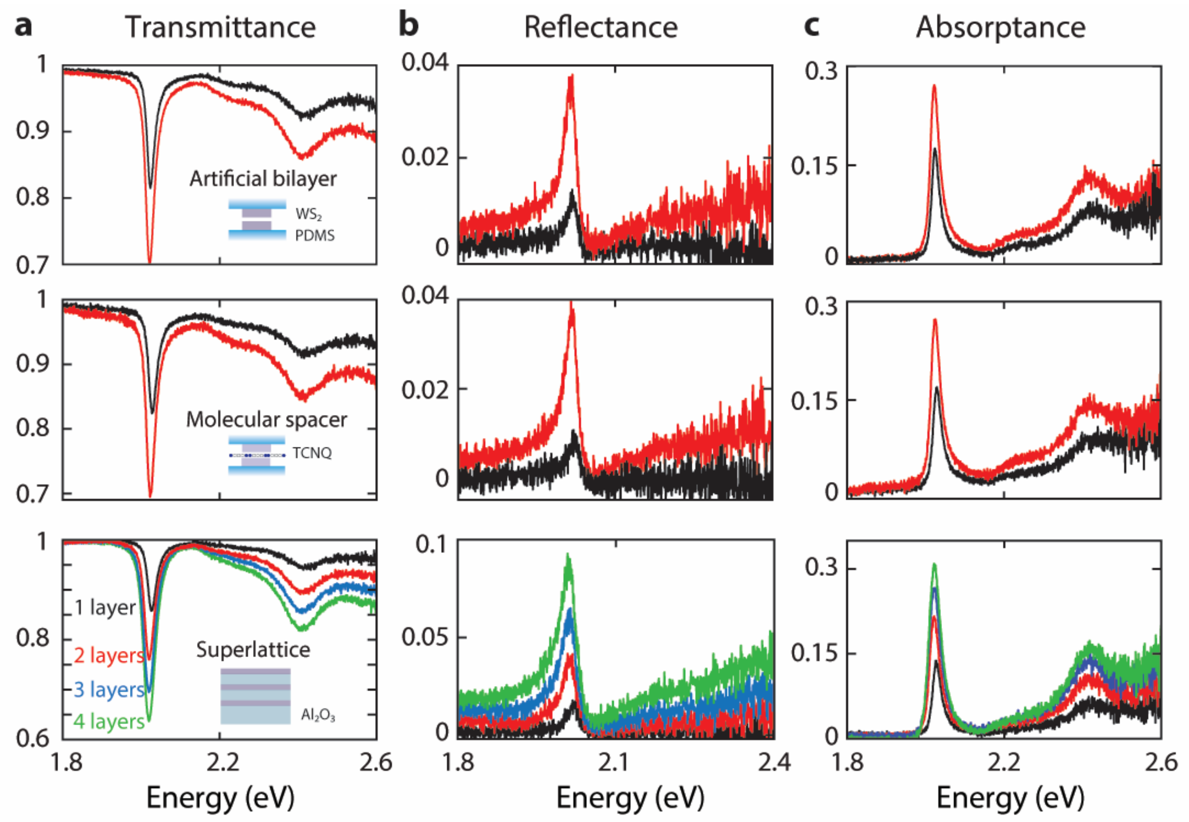

Supporting Figure S2 | Analysis of stacks of WS_2 monolayers with various spacer layers: air (top), molecular (middle), and Al_2O_3 spacers (bottom). **a,** Transmittance. **b,** Reflectance. **c,** Absorptance.

Supporting Section S3. Transfer-matrix method and the maximum absorption limit

We employ the transfer-matrix method (TMM) to determine the transmittance, reflectance, and absorptance of artificial stacks of WS_2 monolayers. The surrounding medium is typically either air or PDMS, depending on the situation. We use the refractive indices of PDMS and air as 1.45 and 1, respectively. For the WS_2 monolayer, we use our experimental permittivity shown in Supporting Figure S3a, which was modeled using a Lorentzian model with 4 oscillators: $\varepsilon(E) = \varepsilon_{background} + \sum_{i=1}^{i=4} f_i/(E_{i,exciton}^2 - E^2 - i\gamma_i E)$, where ε_B is the background permittivity, f_i is the oscillator strength, $E_{i,\ exciton}$ is the resonance exciton energy, $E = \hbar\omega$ is the photon energy, and γ_i is the linewidth of each exciton absorption band.

For each layer, we constructed a transfer matrix (T_{ij}) that describes how a wave passes through the interface from layer i to layer j within the structure. Using the Fresnel equations, we calculated the reflection (R) and transmission (T) coefficients at each interface, which are part of the transfer matrix. We then multiplied these transfer matrices sequentially to obtain the overall transfer matrix for the entire structure: $T_N = T_{1\text{-}2}$ ……. $T_{N\text{-}1}$. From this matrix, we derived the absorptance using $A=1-R-T$. These calculated coefficients and absorptance values were used to plot the optical properties of our structure as a function of wavelength, refractive index, and thickness.

Next, we evaluate the response of a thin film made of layered semiconductor material with thickness d and complex refractive index $n + ik$ sandwiched between two semi-infinite media. We consider only normal incidence. The incident light has a vacuum wavelength λ_0 with its associated wavenumber $q_0 = 2\pi/\lambda_0$. The wavelength-normalized film thickness is $\delta \equiv q_0 d << 1$. Importantly, this thickness must be significantly smaller than the wavelength of light, both within and outside the film, to maintain a wavelength-normalized optical path length $|m\delta|$ below 1. Figure S3b depicts the absorptance for four different film thicknesses as a function of the real and imaginary parts of the refractive index (n and k). The simulation includes thin films of thickness equivalent to a variable number of monolayers, each with the permittivity of a single monolayer and a thickness of 0.618 nm, and a choice of substrate and superstrate: air (n = 1), glass (1.5), and the polymer PDMS (1.42). Notably,

we observe absorptance maxima approaching 50% along the dashed line where $n = k$. For a monolayer in an asymmetric environment, surrounded by PDMS on one side and air on the other, the absorption limit of 50% cannot be reached. Full encapsulation in a symmetric PDMS environment thus increases peak absorption. When the number of layers increases, the absorption maximum moves to smaller n and k values, which are easier to find in realistic materials. Our experimental WS_2 monolayer refractive index is around $5 + 5i$ close to the A-exciton peak represented by the arrows at energy 2.023 eV in the first panel. Therefore, we expect to reach the absorption limit with a stack of 8 monolayers (if they preserve their intrinsic properties after stacking).

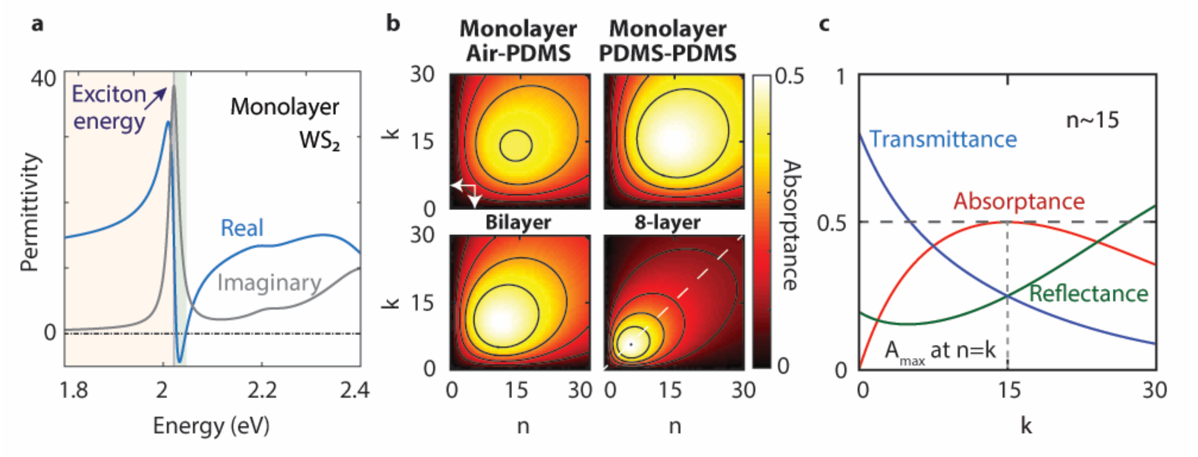

Supporting Figure S3 | Conditions for reaching the maximum absorption limit. **a,** In-plane permittivity of monolayer WS_2, derived by fitting the transmission spectrum using the transfer-matrix method and a 4-Lorentzian permittivity model. **b,** Top: absorptance of a layer with thickness 0.618 nm at $\lambda = 613$ nm for varying real and imaginary parts of the refractive index n and k, for two different superstrates (air with n_{air}=1, or PDMS with n_{PDMS} = 1.42) on a PDMS substrate. Bottom: absorptance of films with a thickness of 2 and 8 times that in the top panels, surrounded by PDMS. **c,** Calculated transmittance, reflectance, and absorptance for a film with monolayer thickness as a function of the imaginary part of the refractive index, k, at a constant n, illustrating the condition for the absorption limit at $n = k$.

Supporting Section S4. Hyperspectral analysis of different spacers

Hyperspectral imaging is a versatile tool for evaluating the homogeneity of monolayer WS_2 samples. By analyzing parameters such as PL intensity, linewidth, and peak energy, we can gain valuable insights into the uniformity and consistency of the samples. In Supporting Figure S4, we compare the spatial distribution of A-exciton peak energy for the three types of monolayer structures studied in the main text fitted from PL hyperspectral images.

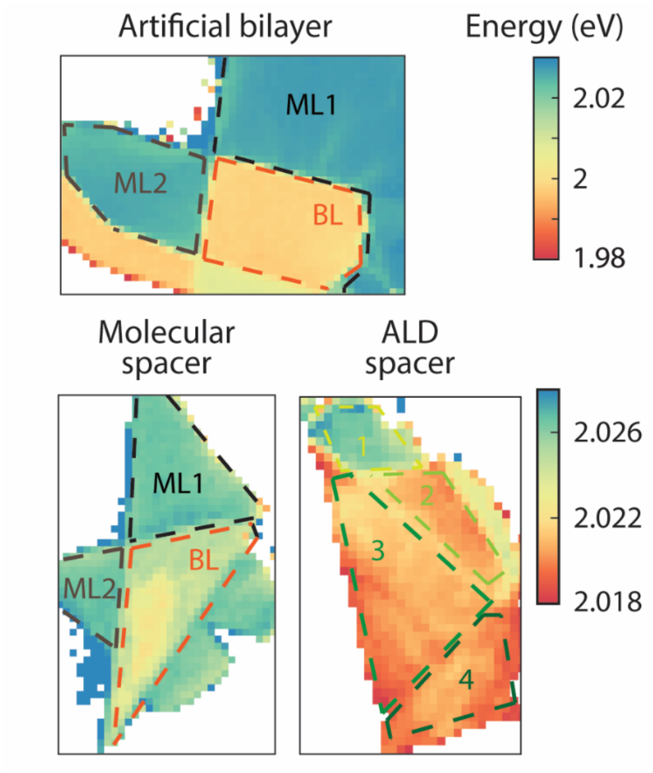

Supporting Figure S4 | Hyperspectral imaging showing the spatial variations of A-exciton peak energy for stacks of WS_2 monolayers with different spacer materials.

Supporting Section S5. Effect of atomic layer deposition on WS2 monolayer

Atomic layer deposition (ALD) enables the creation of uniform and conformal coatings. However, the direct deposition of Al_2O_3 onto monolayer WS_2 via ALD has discernible effects on its optical response, that change its photoluminescence and excitonic behavior. ALD requires solid substrates, as opposed to the polymeric PDMS substrate used elsewhere in this work. Therefore, first we use lifetime measurements to evaluate the monolayer quality before and after depositing it on a different substrate. The photoluminescence decay of WS_2 on a PDMS substrate is slower, indicating longer exciton lifetimes. In contrast, when we transfer a monolayer WS_2 on Al_2O_3-coated amorphous quartz, the lifetime is reduced caused by changes in the electronic environment and increased non-radiative processes (Supporting Figure S5a). On the other hand, in the ALD process, even with a low deposition temperature of 100 °C to minimize damage, the additional defects due to the ALD chemical reaction process negatively affect the quantum efficiency of the monolayer (Supporting Figure S5b).

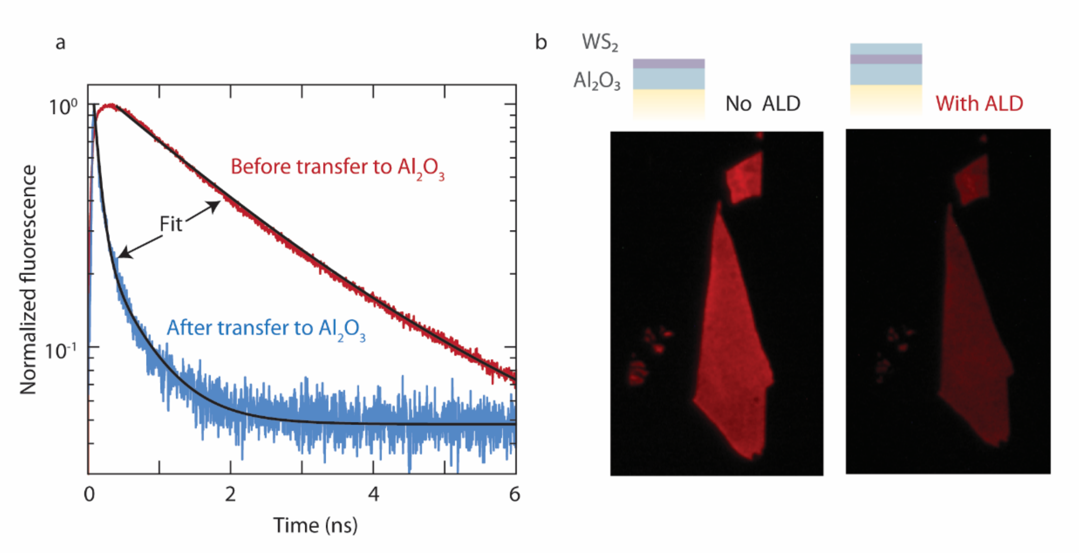

Supporting Figure S5 | Impact of atomic layer deposition on a WS_2 monolayer **a,** Comparison of lifetimes before and after transfer to an Al_2O_3-coated substrate. **b,** Widefield PL images of an as-exfoliated monolayer WS_2 on Al_2O_3/amorphous quartz, and after depositing 2 nm of Al_2O_3 on it using ALD.
